# Human Immune System Mice for the Study of Human Immunodeficiency Virus-Type 1 Infection of the Central Nervous System

**DOI:** 10.3389/fimmu.2018.00649

**Published:** 2018-04-04

**Authors:** Teresa H. Evering, Moriya Tsuji

**Affiliations:** Aaron Diamond AIDS Research Center, An Affiliate of the Rockefeller University, New York, NY, United States

**Keywords:** human immunodeficiency virus, central nervous system, human immune system mice, myeloid cells, HIV-associated neurocognitive disorders

## Abstract

Immunodeficient mice transplanted with human cell populations or tissues, also known as human immune system (HIS) mice, have emerged as an important and versatile tool for the *in vivo* study of human immunodeficiency virus-type 1 (HIV-1) pathogenesis, treatment, and persistence in various biological compartments. Recent work in HIS mice has demonstrated their ability to recapitulate critical aspects of human immune responses to HIV-1 infection, and such studies have informed our knowledge of HIV-1 persistence and latency in the context of combination antiretroviral therapy. The central nervous system (CNS) is a unique, immunologically privileged compartment susceptible to HIV-1 infection, replication, and immune-mediated damage. The unique, neural, and glia-rich cellular composition of this compartment, as well as the important role of infiltrating cells of the myeloid lineage in HIV-1 seeding and replication makes its study of paramount importance, particularly in the context of HIV-1 cure research. Current work on the replication and persistence of HIV-1 in the CNS, as well as cells of the myeloid lineage thought to be important in HIV-1 infection of this compartment, has been aided by the expanded use of these HIS mouse models. In this review, we describe the major HIS mouse models currently in use for the study of HIV-1 neuropathogenesis, recent insights from the field, limitations of the available models, and promising advances in HIS mouse model development.

## Introduction

Infection with human immunodeficiency virus-type 1 (HIV-1) results in CD4+ T cell destruction and progressive debilitation of the immune system (1). Although combination antiretroviral therapy (cART) can effectively suppress HIV-1 RNA to undetectable levels in the peripheral blood (2), the ability of replication-competent HIV-1 to persist in cellular and tissue reservoirs despite suppressive therapy is a barrier to cure (3–5). Penetration of the central nervous system (CNS) by HIV-1 occurs early in infection (6, 7). HIV is postulated to cross the blood–brain barrier (BBB) *via* the infiltration of infected monocytes, CD4+ T lymphocytes (8, 9), or as cell-free virus (10, 11). Resulting CNS immune activation; the infection and activation of monocytes, perivascular macrophages, and resident microglia; and indirect mechanisms are all thought to play a critical role in the pathogenesis of HIV-1 in the CNS (12–16). Early neuropathological characterization of the CNS in those with advanced untreated HIV-1 and HIV-associated dementia (HAD) revealed encephalitis marked by inflammation, microglial activation, astrogliosis, and neuronal loss (17, 18). Use of highly effective cART has significantly reduced the incidence of HAD (19). Nonetheless, HIV-1-associated neurocognitive disorders (HANDs) persist as an important clinical complication of HIV-1 infection in the cART era and can result in an array of cognitive, behavioral, and motor deficits (20). Murine models that mimic human immune systems (HIS) have been extremely valuable tools for the elucidation of a number of pathophysiological mechanisms responsible for HIV-1 CNS pathogenesis. However, no adjunctive therapies for HAND exist beyond cART, and a combination of novel and more physiologically relevant HIS mouse models is now being evaluated to advance our knowledge of the complex immunological and pathological features of HIV-1 neuropathogenesis in the cART era (21, 22).

## Animal Models for Studies of HIV-1 CNS Pathogenesis

Animal models provide an important complementary approach to the study of HIV-1 pathogenesis (23). To varying degrees, these *in vivo* models replicate the intricacies of complex immunological interactions between multiple cell types to an extent not possible *in vitro*. In addition, they are free from many of the experimental constraints imposed by the inaccessibility/limited availability of human tissue (24). Commonly used non-human primate (NHP) animal models include rhesus, pigtail, and cynomolgus macaques that can be infected with a simian or chimeric simian/human immunodeficiency virus. NHP models have provided great insight into HIV-1 neuropathogenesis. In particular, rhesus macaques have been shown to develop HIV encephalitis (HIVE) and microglial infection (25), and a highly neurovirulent (although not physiologic) challenge model has been developed in pigtail macaques (26). However, studies using NHPs are limited by high cost, special housing requirements, and small experimental groups. In response to these constraints, small animal models of human disease have been developed and widely employed (27). However, their ability to recapitulate human disease can be limited as some important human pathogens (including HIV) display tropism unique to humans (28, 29). The transgenic expression of select HIV-1 proteins such as HIV-1 envelope and trans-activator of transcription, human receptors and co-receptors in mice result in animals with a broad range of HIV-1-related pathologies (30–34). These include a spectrum of neurotoxicity, defective neurogenesis, and glial abnormalities in mouse CNS that resemble those seen in the brains of HIV-1-infected humans (35–39). Although these transgenic models mirror specific components of the pathophysiological effects of select HIV-1 proteins on the CNS (40), as well as some of the cognitive and behavioral features of HAND (41), they are unable to model critical aspects of HIV-1 CNS infection in the human host, such as viral CNS invasion (42). For these reasons, the use of small animal models that can more accurately mimic the HIS is of great value.

## Human Immune System (HIS) Mouse Models for the Study of HIV-1

In contrast to transgenic or chimeric mice, the creation of mice with human immune system components (HIS mice) provide an *in vivo* environment that allows for the study of HIV-1 and its interaction with cells of the human immune system (24). HIS mouse production initiates with the choice of an immunodeficient mouse strain that can accommodate the engraftment of human cells and tissues without rejection (43). Early immunodeficient mice used for human tissue or cell xenografts included “nude” mice, which lack mature CD4+ and CD8+ T cells (44) and severe combined immunodeficiency (SCID) mice, which harbor a mutation in the protein kinase, DNA-activated, catalytic polypeptide gene (Prkdcscid) and lack mature T and B cells (45). The ability of these strains to support long-term, systemic reconstitution with human cells were, however, limited by relatively high residual levels of innate immune responses, such as those mediated by natural killer (NK) cells resulting in the rejection of human bone-marrow allographs (46). Improved levels of immunodeficiency were found in strains lacking mature B and T lymphocytes due to disruptions in the recombination-activating genes Rag1 and Rag2 (47, 48), that were further augmented in mice also harboring a complete null mutation of the common cytokine receptor γ chain (IL2Rγ, or γc), resulting in the absence of mouse NK cells (49–51). As a result, modern HIS mouse models are typically produced by engrafting human hematopoietic stem cells (hHSCs), human peripheral mononuclear cells, and/or human tissues into these highly immunodeficient strains following their preconditioning with sublethal irradiation or chemotherapy. The main platforms in use include NSG (NOD-scid IL2Rγnull and NOD.Cg-PrkdcscidIL-2Rγtm1Wjll/Sz) (52), NRG (NOD-Rag1−/−IL2RγC-null), NOG (NOD.Cg-Prkdcscid IL-2Rγtm1Sug), and BRG (BALB/c-Rag2null IL-2Rγnull) strains (24, 53). Although important differences in the extent of humanization and functional quality of the populating human cells exist between models, multilineage reconstitution with hHSCs can include all major human lymphocyte classes (CD4+ and CD8+ T cells, B cells, and NK cells) as well as various myeloid cells (monocytes, macrophages, and dendritic cells). In those strains of mice that support human T-cell development when transplanted with human CD34+ hHSCs, T cell maturation occurs in the murine thymus (52, 54, 55). When humanized mice are engineered by implanting human thymus and liver tissue, developing T cells are educated on human thymic epithelial cells, allowing for restriction by human leukocyte antigens (HLAs) I and II (56, 57). The bone marrow–liver–thymus (BLT) mouse model, which combines the implantation of fetal liver and thymus under the kidney capsule of NOD/SCID, NSG, or C57BL/6 Rag2−/− IL2γ−/− mice, along with the transplant of autologous CD34+ hHSCs is the most complete and well explored (58–60). The technical demands of this system are associated with considerable expense, and the need to surgically implant each mouse can result in significant variation in HIS repopulation (61). However, with their strong lymph node and intestinal reconstitution, BLT mice are particularly useful for the study of HIV-1 infection at mucosal surfaces (62–64). Modern HIS mouse models provide stable human cellular reconstitution that can support HIV-1 replication in the peripheral blood and multiple organs (27), allowing them to provide insights into many aspects of HIV-1 biology including viral life cycle and innate and adaptive immune responses to HIV-1 (59, 62, 65–68). Viral suppression with clinically relevant cART (69–73) has been demonstrated in HIS mice, and they have proven effective for the investigation of multiple immune-based approaches for the *in vivo* control of viral replication and elimination of HIV-infected cells (74–78).

## HIS Models for the Study of HIV-1 Neuropathogenesis and Response to Treatment

Early neuroAIDS mouse models involved the generation of HIVE through the direct injection of human microglia or macrophages into the brain of SCID mice (79, 80). While the resultant SCID-HIVE model recapitulates some of the neuropathological features of human HIVE, these approaches are traumatic and result in xenoreactivity induced-inflammation through the artificial insertion of human cells into a foreign mouse cellular environment (81). Despite these caveats, studies investigating the impact of cART in this model have demonstrated reductions in neuropathological features of HIVE including decreased astro- and micro-gliosis and reductions in HIV-1 brain viral loads (82–84). Subsequent development of the humanized mouse model, in which NSG mice are engrafted with CD34+ hHSCs (CD34+-NSG mice), has allowed for more detailed, prolonged studies of HIV-1 CNS infection and neurodegeneration in the context of unchecked HIV-1 replication (85). Systemic HIV-1 infection in this model is characterized by low CNS viral burdens and the transmigration of HIV-infected human monocytes and macrophages into the mouse CNS. These human cells localize predominantly to the meninges, perivascular spaces, and, to a lesser extent, brain parenchyma (85–88). Regional activation of resident murine microglia and astrocytes, neuroinflammation, and neurodegeneration are also among the salient findings in this model (85, 86, 89). Some of these changes were reversible by long-acting nanoparticle-based cART (90). More recently, pre- and post-infection dosing with a novel sonic hedgehog mimetic was found to increase BBB integrity in acutely infected CD34+-NSG mice, resulting in decreased leukocyte extravasation into CNS during and pathologic evidence of neuroprotection (91). Finally, a simplified HIS model generated by the intraperitoneal (IP) injection of human PBMCs into non-irradiated NSG mice (NSG-huPBL) has recently been described (92). In this model, IP challenge with HIV-1 resulted in systemic viral infection and CNS invasion with infected CD4+ T cells. The presence of neuropathology—characterized by neurodegeneration, activated microglia, and astrocytes—was found to be dependent on the infecting viral strain (93). A brief summary of currently available HIS mouse models with published data on HIV-1 CNS infection can be found in Table 1.

**Table 1:** HIS mouse models with published studies of human immunodeficiency virus-type 1 (HIV-1) infection of the central nervous system (CNS).

HIS mouse model (reference)	Method of generation	Salient CNS findings in response to HIV-1 infection
Severe combined immunodeficiency (SCID)–HIV encephalitis (HIVE) (79–84)	Direct injection of HIV-1-infected human microglia or macrophages into the brain of SCID mice	Measurable HIV-1 brain viral load and neuropathological features of HIVE including astrogliosis and microgliosis. Reduction in CNS pathology in response to combination antiretroviral therapy (cART).

NSG-huPBL (93)	Intraperitoneal injection of human donor PBMCs into non-irradiated NSG mice	HIV-1-infected human CD4+ T cells present in meninges and cortex of infected animals. Appearance of neurodegeneration, microgliosis, and astrogliosis dependent on infecting viral strain.

CD34+-NSG (85, 86, 89–91, 105)	NSG mice transplanted with human CD34+ hematopoietic stem cells (hCD34+)	Low CNS viral burdens, transmigration of HIV-infected human monocytes and macrophages into the mouse CNS, regional activation of resident murine microglia and astrocytes, neuroinflammation, and neurodegeneration. Reduction in CNS pathology with long-acting nanoparticle-based cART. Increased blood–brain barrier integrity in acutely infected CD34+-NSG mice and decreased leukocyte extravasation into CNS following treatment with a novel sonic hedgehog mimetic.

CD34+-NSG (+hNPC) (133)	NSG mice transplanted with hCD34+ combined with intraventricular injection of neural progenitor cells	Detection of human glia in diverse brain regions of HIS mice including periventricular areas, white matter tracts and brain stem. Mice infected with HIV-1 display glial transcriptional signatures and viral defense signaling pathways that mirror human disease.

Myeloid-only mice (60)	NOD/SCID mice transplanted with hCD34+	HIV-1 DNA and RNA as well as macrophages expressing HIV-1 p24 detected in the brains of infected animals.

DRAG (121)	NRG mice expressing human leukocyte antigen (HLA) class II (DR4) transplanted with HLA-matched hHSC	HIV-1 replication in brain following mucosal infection.

## HIS Models in Elucidating the Role of Myeloid Cells in HIV-1 CNS Persistence

Monocytes and macrophages can be infected with HIV-1 both *in vitro* and *in vivo* (94–96). However, the question of whether cells of myeloid lineage serve as true HIV-1 reservoirs in the context of suppressive cART remains of great interest (97). This question is central to the study of HIV-1 persistence in the CNS as perivascular monocyte-derived macrophages and parenchymal microglia are the most important cellular targets of HIV-1 in the CNS (98), and infection of these cell types is critical to HIV-1 CNS pathogenesis and HAND (99). Recent evidence suggesting that macrophages may become positive for viral DNA through the capture and phagocytosis of infected CD4+ T cells implies a mechanism of infection distinct from virological synapse formation and furthers the debate (100, 101). Recent study in the T cell only mouse in which implantation of autologous human fetal liver and thymus under the kidney capsule of an NSG mouse results in systemic reconstitution almost exclusively with human T cells predictably demonstrates the development of latent T cell reservoirs of HIV-1 (102). Complementary studies by Honeycutt et al. in myeloid-only mice (MoM) in which NOD/SCID mice transplanted with CD34+ hHSCs are reconstituted with human myeloid and B cells in the absence of human T cells have proven informative. Using this novel HIS model, Honeycutt et al. demonstrated that macrophages can support efficient HIV-1 replication *in vivo* in multiple compartments in the absence of T cells following infection with certain macrophage-tropic (M-tropic) HIV-1 strains such as HIV-1 ADA. HIV-1 DNA and RNA as well as macrophages expressing HIV-1 p24 were detected in the brains of infected MoM (60). In addition, cessation of suppressive cART in MoM resulted in measurable *in vivo* viral rebound after 7 weeks (103) supporting infection of long-lived tissue macrophage populations (104). Another recent study in CD34+-NSG mice infected with M-tropic HIV-1 found evidence for CD14+CD16+ monocyte/macrophage cells with HIV-1 RNA and integrated proviral DNA in the spleen and bone marrow. Consistent with previous reports in this model, viral RNA was detected in the brains in a few animals at low copy numbers (105). As a result, HIS mouse models have proven utility in defining cellular sites for HIV-1 infection and hold promise for further elucidating the viral dynamics of the establishment and recrudescence of potential CNS-based HIV-1 reservoirs.

## Current Challenges and Advances in HIS Models for the Study of HIV-1 in the CNS

Although they represent powerful research tools, limitations to the use of HIS mice for the *in vivo* study of HIV-1 exist. HIS models do not perfectly recapitulate human hematopoiesis and can display a relatively short lifespan, particularly after the approximately 8- to 18-week period needed for appropriate engraftment (43). Variability in the efficiency of human cell engraftment is an important challenge to robust experimentation. In addition, despite the fact that most HIS mouse models have demonstrated highly effective adaptive T-cell immune responses, the majority of models display an absence of species-specific human cytokines and impaired B-cell function and humoral immune responses (55, 106–108). This is important, as one proposed mechanism for the pathology induced in the CNS in response to HIV-1 infection is an abnormal cytokine/chemokine response (16). Another important limitation of currently available HIS mouse models is the frequent development of graft-versus-host disease (GVHD), characterized by multiorgan lymphocytic infiltration and sclerosis in the weeks following hHSC transplant (109). This is an important limitation to the study of HIV-1 in the CNS in particular, as longer-term experiments are necessary to demonstrate productive infection of the CNS by HIV-1 and CNS pathology in animals naïve to and under cART and/or putative adjunct therapeutics. Several research groups are working to improve the functionality of HIS mouse models in response to these limitations. Lavender et al. have described the evaluation of GVHD-resistant triple knockout (TKO) mice, which lack CD47 in addition to Rag 1 and IL2rg. These TKO-BLT mice reportedly remained healthy for 45 weeks post-humanization and could be virally suppressed on cART (110). Additional efforts to improve HIS mouse platforms have included the depletion of endogenous mouse macrophages (111) and the development of strains expressing human cytokines for improved human NK-cell development (112, 113). HIS models with improved development of HLA-restricted human T cells have been achieved through engraftment of HLA-matched hHSC into immunodeficient mice with transgenic expression of human HLA molecules (114). Huang et al. have reported the development of a novel HIS mouse model utilizing recombinant adeno-associated virus-based gene transfer technologies (115) to introduce genes encoding HLA-A2/DR and selected human cytokines into NSG mice. The ability of this resultant HIS mouse model to endogenously encode for human MHC constitutively during its lifespan and key human cytokines during development of lymphoid and myeloid progenitor cells allows for an accurate recapitulation of many aspects of the human immune system. This is reflected in highly functional human CD4+ and CD8+T-cell and B-cell responses (116, 117) as well as the successful reconstitution of human monocytes (CD14+) and macrophages (CD14+/CD11b+) (117). These HIS mice can be productively infected with HIV-1 (118) and have the ability to secrete measurable human IFN-γ, IL-2, CCL3, and IL-1β *in vivo* in response to parasitic and viral pathogens (117–120). With high rates of engraftment and low rates of GVHD, this model can be a useful tool for the study of potentially important viral reservoirs of HIV-1 in the CNS. In a similar vein, Kim et al. have recently reported the use of immunodeficient mice expressing HLA class II (DR4) (DRAG mice) engrafted with HLA-matched hHSCs to study early HIV-1 infection. The authors report HIV-1 replication in various tissues, including bone marrow, lymph nodes, and the brain, which on day 21 following mucosal infection, was the last tissue examined to become HIV-1 viral RNA positive (121). Finally, infiltrating human myeloid cells and lymphocytes have been demonstrated in the brains of HIV-1 infected HIS mice (85). However, the generation of models harboring functional human myeloid cells in percentages approximating those seen in humans has been challenging. In several HIS platforms, strategies to improve human myeloid cell reconstitution include the administration of exogenous human Flt3 ligand (122), exogenous delivery of human granulocyte-macrophage colony-stimulating factor (GM-CSF) and IL-4 (123, 124), and human GM-CSF and IL-3 knock-in (125, 126).

Limitations of HIS mouse models that are of unique interest to the study of HIV-1 in the CNS exist as well. Common to all HIS mouse models is the absence of human microglia in the CNS (127)—a major deficiency as microglia represent one of the most important cellular targets of HIV-1 in the brain (98). Unfortunately, engrafted CD34+ hHSCs are unlikely to repopulate human microglial cells within the brains of HIS mice, as microglial cells are derived early during development from yolk sack precursors (127). Additionally, human glia (astrocytes and oligodendrocytes)—the most abundant cell types in the human CNS—are absent in the majority of HIS mouse models (128). As a result, these platforms are unable to recapitulate innate glial cell responses resulting from the complex interactions between human glia and infected mononuclear phagocytes during progressive HIV-1 infection (129, 130). In response, several groups have attempted to reconstitute HIS mouse brain with neonatally transplanted human glial progenitor cells (131, 132). Following such interventions, Li et al. reported the detection of human glia in diverse brain regions of HIS mice including periventricular areas, white matter tracts, and brain stem. RNA-sequencing in the selected brain regions of such mice infected with M-tropic HIV-1 reportedly display glial transcriptional signatures and viral defense signaling pathways that mirror human disease (133–136). Although this approach does not repopulate the brain with human microglia, such experimental improvements are welcome and will allow for the improved modeling of human HIV-1 CNS neuropathological disease.

## Conclusion

Human immune system (HIS) mouse models have proven to be extremely valuable tools for the study of HIV-1 infection of the CNS, its resulting neuropathology and the potential for HIV-1 persistence in this immunologically privileged compartment. As with all model systems, experimental and biologic limitations exist. These include the absence of human CNS cell types that in response to HIV-1 invasion play key roles in the development of the neuroinflammatory milieu and impaired immune, glial, and neural cell functions leading to HAND. However, model improvements are ongoing, with the general aims of preventing GVHD and enhancing the levels, reproducibility, and quality of human immune cell reconstitution. The rational evolution of these models will continue to foster authentic human immune responses in HIS mouse models and will further facilitate development of diagnostic, novel therapeutic, and viral eradication strategies for HIV-1 in the CNS.

## Author Contributions

TE and MT contributed to the conception, writing, and discussion of this review manuscript. TE wrote the initial draft of the manuscript. The final version of the manuscript was approved by both authors.

## Conflict of Interest Statement

The authors declare that the research was conducted in the absence of any commercial or financial relationships that could be construed as a potential conflict of interest.
